# Hypermongone C Accelerates Wound Healing through the Modulation of Inflammatory Factors and Promotion of Fibroblast Migration

**DOI:** 10.3390/molecules24102022

**Published:** 2019-05-27

**Authors:** Sara E. Moghadam, Mahdi Moridi Farimani, Sara Soroury, Samad N. Ebrahimi, Ehsan Jabbarzadeh

**Affiliations:** 1 Department of Chemical Engineering, University of South Carolina, Columbia, SC 29208, USA; eslambol@mailbox.sc.edu; 2 Department of Phytochemistry, Medicinal Plants and Drug Research Institute, Shahid Beheshti University, GC, Evin, Tehran 1983969411, Iran; m_moridi@sbu.ac.ir (M.M.F.); s_ebrahimi@sbu.ac.ir (S.N.E.); 3 Department of Phytochemistry, Faculty of Science, Golestan University, Gorgan 49138157559, Iran; sarasoroury@gmail.com; 4 Biomedical Engineering Program, University of South Carolina, Columbia, SC 29208, USA

**Keywords:** wound healing, immunomodulation, angiogenesis, *Hypericum scabrum*, hypermongone

## Abstract

The physiology of wound healing is dependent on the crosstalk between inflammatory mediators and cellular components of skin regeneration including fibroblasts and endothelial cells. Therefore, strategies to promote healing must regulate this crosstalk to achieve maximum efficacy. In light of the remarkable potential of natural compounds to target multiple signaling mechanisms, this study aims to demonstrate the potential of hypermongone C, a polycyclic polyprenylated acylphloroglucinol (PPAP), to accelerate wound closure by concurrently enhancing fibroblast proliferation and migration, promoting angiogenesis, and suppressing pro-inflammatory cytokines. This compound belongs to a family of plants (*Hypericum*) that traditionally have been used to treat injuries. Nevertheless, the exact biological evidence to support the claims is still missing. The results were obtained using a traditional model of cell scratch assay and endothelial cell tube formation, combined with the analysis of protein and gene expression by macrophages. In summary, the data suggest that hypermongone C is a multi-targeting therapeutic natural compound for the promotion of tissue repair and the regulation of inflammation.

## 1. Introduction

Skin tissue repair and wound healing is a very complex biological process which is controlled by different cell types including macrophage immune cells, dermal fibroblasts, epidermal keratinocytes, and endothelial cells working in concert to regenerate damaged skin. Wound healing comprises four major overlapping phases: (1) hemostasis or blood clotting; (2) inflammation, which is associated with the migration of macrophages and immune cells to the wound bed to invade pathogens and clean the wound [1,2]; (3) proliferation and tissue formation, which involves the proliferation and migration of fibroblasts and the development of new blood vessels (angiogenesis); and (4) tissue remodeling. Upon injury, different cell types are recruited to the wound site in a timely manner to secrete growth factors and cytokines in response to external pathogens and tissue rupture to initiate the healing process [3,4,5].

Inflammation is regulated by macrophages, which play a distinct role in each phase of healing due to phenotype changes in response to environmental stimuli [6]. In the first stage of healing, inflammatory cells migrate to the wound site and differentiate into classically polarized M1 macrophages, which are associated with tissue injury and inflammation. In the inflammatory phase of wound healing, M1 macrophages remove apoptotic cell debris and secrete inflammatory cytokines and growth factors, such as tumor necrosis factor alpha (TNF-α), interleukin-6 (IL-6), and vascular endothelial growth factor (VEGF). In the second phase of healing, macrophages are differentiated into the activated anti-inflammatory M2 subtype to promote cell proliferation and migration, and to overcome inflammation [2,7]. If the inflammatory response is sustained, the wound becomes chronic, which leads to impaired healing [2,8].

One of the most important steps in wound closure is the ability for cells to migrate into the wound bed. The tissue formation stage of wound healing is performed by the proliferation and migration of dermal fibroblasts, which are themselves stimulated by macrophages and growth factors. Fibroblasts are responsible for producing the extracellular matrix (ECM) and collagen, which ultimately form the granulation tissue [6,9]. This matrix functions as tissue support and facilitates the migration of other cells, such as endothelial cells, macrophages, and fibroblasts [10,11]. Fibroblasts are differentiated into myofibroblast, and collagen type III is replaced by collagen type I at the late stage of healing, which leads to the maturation of the newly formed tissue [12,13,14].

Angiogenesis or new blood vessel formation is another process that occurs during wound healing and is initiated immediately after tissue injury. Angiogenic factors, such as VEGF released from damaged endothelial cells and macrophages, induce endothelial cells to develop micro-vessels from pre-existing vessels. Capillary tubes extend branches to transfer oxygen and nutrients to the granulation tissue, which is required for successful tissue regeneration and wound closure [15,16]. One of the characteristics of delayed healing and chronic wounds is the failure of vascularization and blood circulation [17,18,19].

There is a vital need for new drugs that regulate different phases of healing simultaneously. Historically, natural compounds have been used in treating wounds due to their antioxidant, anti-inflammatory, and antimicrobial properties. Natural compounds are uniquely positioned to play a regenerative role in wound repair by targeting multiple phases of healing, such as the modulation of cytokine and growth factor secretion, as well as the induction of cell proliferation and migration [20,21]. Among natural compounds, one family that has attracted significant attention is *Hypericum* [22,23]. *Hypericum* belongs to the family of Gutiferaceae (also known as Hypericaceae), a genus that features an abundance of polycyclic polyprenylated acylphloroglucinols (PPAPs) [24]. Their bioactivities are attributed to the complex and unique structure of acylphloroglucinol [25]. Medicinal plants from this genus have been used in traditional medicine due to their antibacterial, neuroprotective, and anti-inflammatory activities [26,27]. One of the most important and well-known species, *Hypericum perforatum* (St. John’s wort), has demonstrated significant wound healing effects in skin injuries and is considered safe for use [28]. Additionally, local application of *H. perforatum* extract in combination with neem oil (Holoil^®^) has shown anti-inflammatory effects in patients undergoing radiotherapy treatment [29]. However, species of this genus are rarely studied, suggesting further investigation is warranted.

To address this knowledge gap, in this study, we elucidated the wound healing effects of hypermongone C, a compound isolated from *Hypericum scabrum*. This was assessed by monitoring the migration and proliferation of dermal fibroblasts, tube formation of endothelial cells, and cytokine expression of pro-inflammatory macrophages in response to varying doses of hypermongone C. Our findings demonstrate the enormous potential of this compound to regulate the interaction among multiple components of the regenerative process and accelerate wound closure effectively.

## 2. Results

### 2.1. Hypermongone C Identification

**Figure 1:**
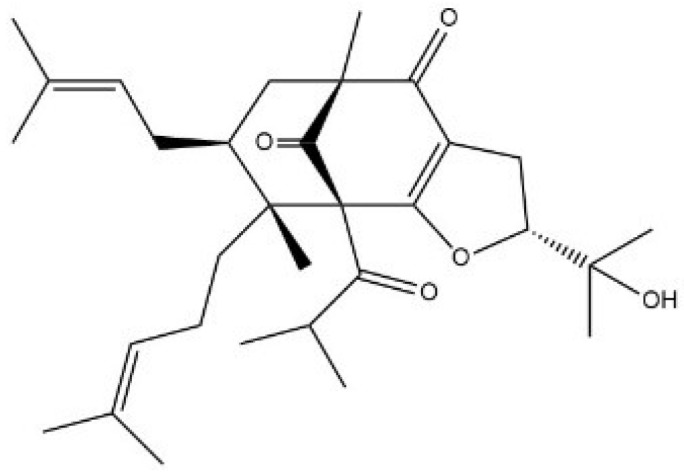
The structure of hypermongone C, a polycyclic polyprenylated acylphloroglucinol.

Using time of flight mass spectrometry (TOF-MS) in the positive ion mode, we determined that the compound isolated from *H. scabrum* had a molecular formula of C_31_H_46_O_5_ ([M + H]^+^ ion at *m*/*z* 499.3414, corresponding to C_31_H_47_O_5_). The structure (Figure 1) was assigned using nuclear magnetic resonance (NMR) (Figure S1 and Table S1) and high-resolution mass spectrometry (Figure S2). We identified the compound as a PPAP based on our analysis of 1D and 2D-NMR results, having a furan ring, two isoprenyl groups, tertiary and quaternary methyl, and three carbonyl groups connected to different parts of the structure, which are the key features of this class of compounds. The comparison of NMR chemical shifts and MS data with reported values and known compounds in the literature confirmed our compound as hypermongone C [30]. The heteronuclear single quantum coherence (HSQC) and heteronuclear multiple bond correlation (HMBC) correlations established the bicyclic structure (Figure S1).

### 2.2. Effect of Hypermongone C on Cell Proliferation and Viability

**Figure 2:**
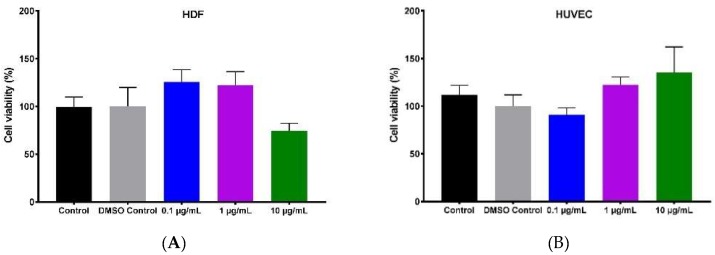

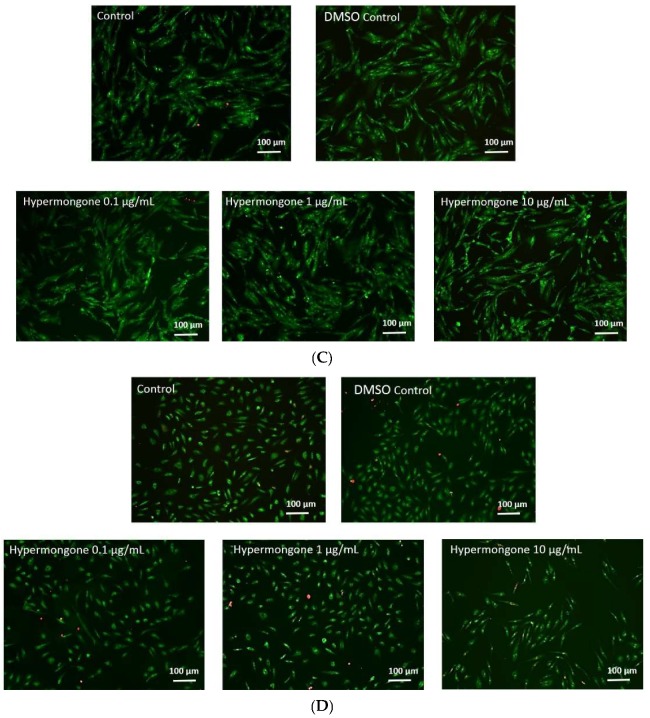
Concentration-dependent cell proliferation of (**A**) human dermal fibroblasts (HDFs) in response to various concentrations of hypermongone C after 24-h exposure, calculated using the MTS assay. No toxicity was observed in the range of 0.1 and 1 µg/mL when compared to the control (untreated cells in growth media). (**B**) The percentage viability of human umbilical vein endothelial cells (HUVECs) in response to 8-h incubation with various concentrations of hypermongone C did not show toxicity by the MTS assay. (**C**) The LIVE/DEAD^®^ assay of HDFs and (**D**) HUVECs confirmed the non-toxic effect of hypermongone C on the cells.

MTS ((3-(4,5-dimethylthiazol-2-yl)-5-(3-carboxymethoxyphenyl)-2-(4-sulfophenyl)-2*H*-tetrazolium) assay was used as an indicator of metabolic activity which indirectly measures cell proliferation and viability. We performed the MTS assay to determine the cell viability of human umbilical vein endothelial cells (HUVECs) and primary human dermal fibroblasts (HDFs), cultured in a 96-well plate and treated with different concentrations of hypermongone C (0.1, 1, and 10 µg/mL) for 24 h. HDF cells demonstrated higher proliferation at concentrations of 0.1 and 1 µg/mL as compared to the non-treated cells. Hypermongone C at the concentration of 10 µg/mL moderately inhibited cell growth (Figure 2A). HUVECs treated with different concentrations of hypermongone C (0.1, 1, and 10 µg/mL) for 8 h demonstrated no toxicity, and concentrations of 0.1 and 1 µg/mL were used for further experiments (Figure 2B). To confirm the cell viability of HDFs and HUVECs treated with different concentrations of hypermongone C, we performed the LIVE/DEAD^®^ assay. Following 24-h incubation, hypermongone C did not induce toxicity in HDFs and HUVECs, and the live cells were visualized as green (Figure 2C,D).

### 2.3. Hypermongone C-Induced Cell Migration

**Figure 3:**
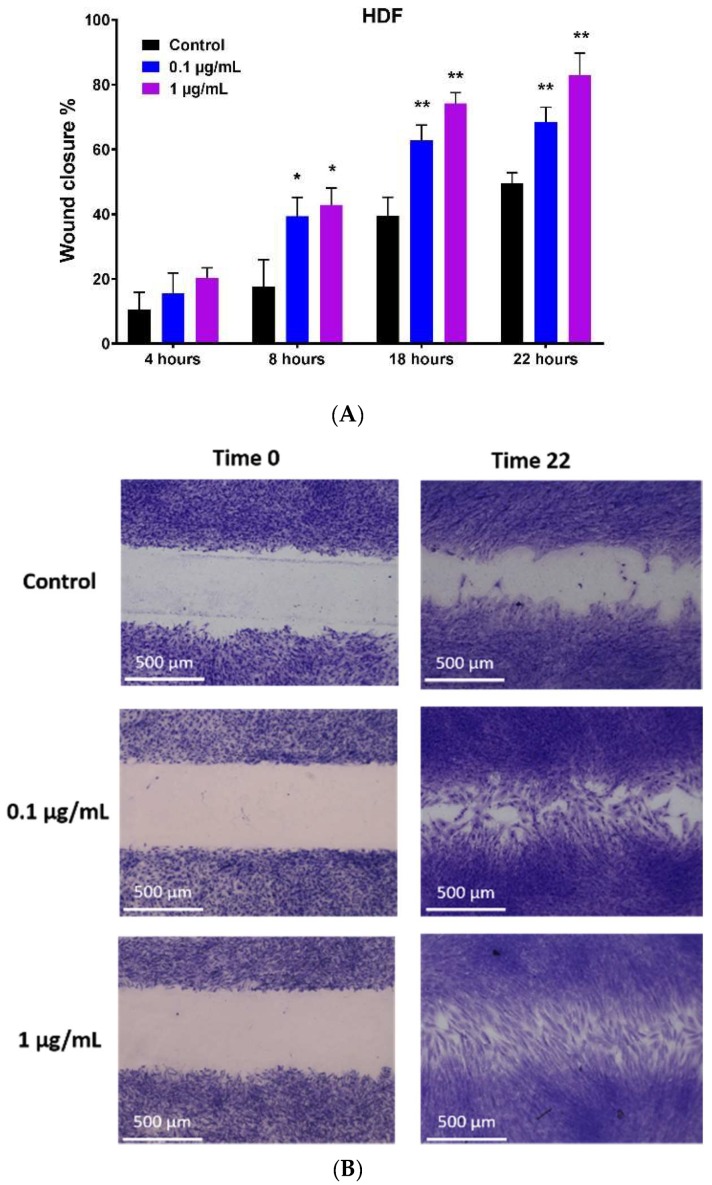
Migration assay of hypermongone C. (**A**) Wound closure percentage of HDFs after different time intervals of exposure to different concentrations of hypermongone C. The compound at a concentration of 1 µg/mL demonstrated the highest migration over 22 h. Multiple t-tests were performed using Graph-Pad Prism 7.03 to determine the significance between each experimental group and control (* *p* ≤ 0.05 and ** *p* ≤ 0.01). (**B**) Representative images of each treatment group after 22 h.

The effect of hypermongone C on the migration of HDFs to a cell-free gap was investigated using a scratch assay. Cells were treated with non-toxic concentrations of hypermongone C at 0.1 and 1 µg/mL for 22 h. We observed that hypermongone C induced the migration of cells toward the cell-free gap (Figure 3). The percentage of gap closure was imaged at different time points, and there was a significant concentration-dependent closure effect compared to the control or untreated cells. At the early time point of 4 h, we did not observe any significant difference in cell migration among different groups. However, over 8 h, the number of cells that migrated toward the scratch was higher compared to the control for both concentrations. Wound closure was almost two times faster for concentrations of 0.1 and 1 µg/mL over 18-h incubation. This effect was also concentration-dependent; as the concentration of the compound increased, migration became faster. At the 22 h time point, 1 µg/mL hypermongone C treatment showed a remarkable 80% closure, which was significantly higher than the control (45%).

### 2.4. Effect of Hypermongone C on Angiogenesis

**Figure 4:**
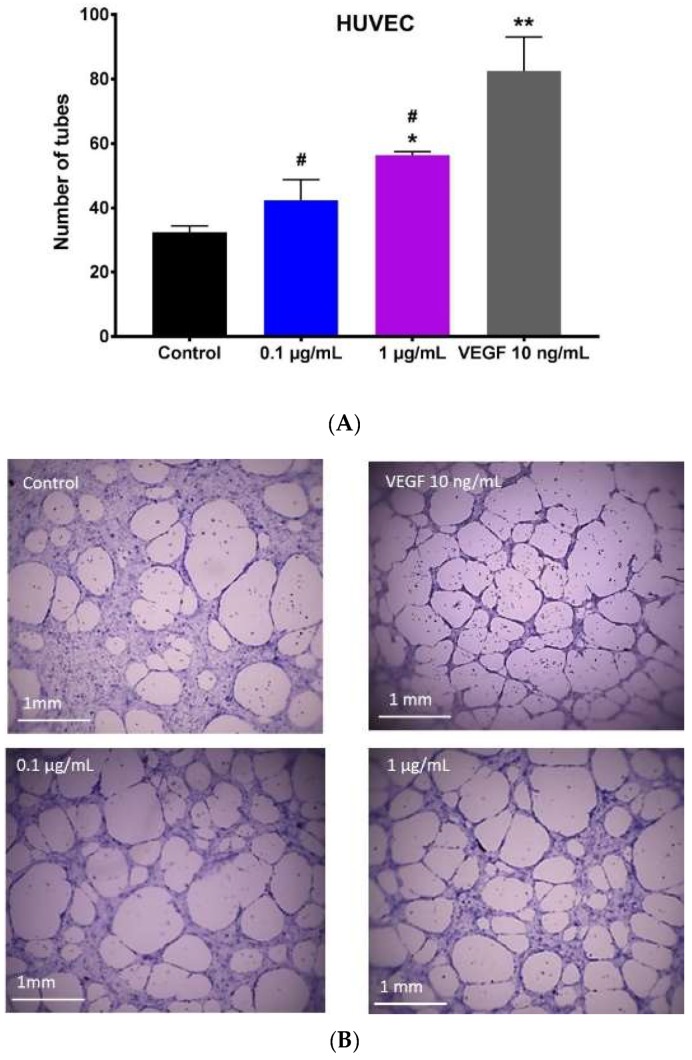
(**A**) The average number of tubes formed among HUVECs after 8 h of incubation on growth factor reduced BD matrigel, in which * represents a significant difference (*p* ≤ 0.05 and ** *p* ≤ 0.01) from the control group, which was calculated using Graph-Pad Prism 7.03 to determine the significance between each experimental group and control. ^#^ represents a significant difference (*p* ≤ 0.05) among treated groups and VEGF positive control. (**B**) Representative images are shown for the treatment with growth media alone, 0.1 µg/mL, and 1 µg/mL hypermongone C. Fewer tubes were formed in the group including untreated cells, with many of them remaining as individual single cells.

To study the effect of hypermongone C on angiogenesis, we seeded HUVECs on a matrigel basement membrane as a substrate. Over 8-h incubation with the compound at concentrations of 0.1 and 1 µg/mL, we observed the cells to proliferate and migrate to form tubular networks. The number of tubes was counted manually in triplicate for the groups treated with culture media alone or with different concentrations of hypermongone C (Figure 4A). Cells treated with the compound showed a higher level of tubing network as compared to the untreated control, in which many of the cells remained scattered (Figure 4B). There was a significant difference in the number of tubes formed for the hypermongone C treatment concentration of 1 µg/mL compared to the control, clearly indicating the ability of this compound to induce the type of tube formation necessary for wound healing. VEGF is an ideal positive control for the tube formation; however, the number of tubes at concentrations 0.1 and 1 µg/mL did not reach that of the group containing VEGF.

### 2.5. Effect of Hypermongone C on TNF-α, IL-6, and VEGF Production

**Figure 5:**
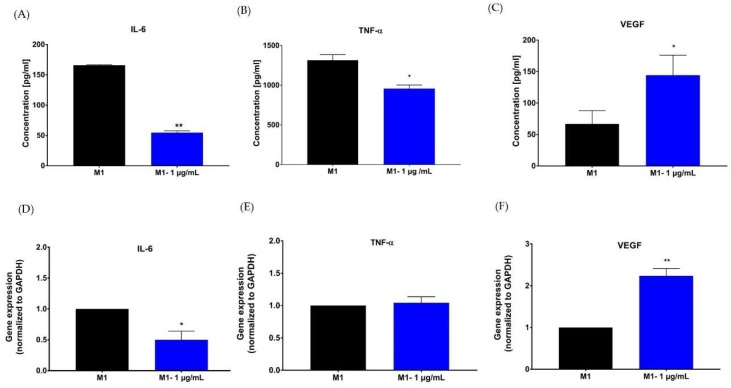
The influence of hypermongone C on cytokine expression in culture media was determined for (**A**) interleukin (IL)-6, (**B**) tumor necrosis factor (TNF)-α, and (**C**) vascular endothelial growth factor (VEGF) by enzyme-linked immunosorbent assay (ELISA) assay. Gene expression of (**D**) IL-6, (**E**) TNF-α, and (**F**) VEGF by M1 macrophages was determined after 24 h treatment hypermongone C using quantitative real-time PCR. Gene expression was normalized to the housekeeping gene GAPDH (internal control) and the control group (non-treated M1 macrophages (2^−ΔΔC^)). * *p* ≤ 0.05 and ** *p* ≤ 0.01 as compared to the control group.

We assessed the effect of hypermongone C on the macrophage inflammatory response using enzyme-linked immunosorbent assay (ELISA) and quantitative real-time polymerase chain reaction (qRT-PCR) assays. Differentiated THP-1 macrophages (M0 macrophage) were polarized to the M1 pro-inflammatory phenotype. The effect of hypermongone C treatment on the lipopolysaccharide (LPS) and interferon-gamma (IFN-γ) polarized macrophages demonstrated that expression of both inflammatory markers IL-6 and TNF-α were significantly decreased compared to the non-treated M1 macrophage (Figure 5A,B). Hypermongone at 1 µg/mL over 24-h treatment downregulated the expression of cytokines IL-6 and TNF-α by 3-fold and 1.5-fold, respectively. This reduction was more prominent for IL-6 than for TNF- α. The compound also caused a drastic increase in VEGF growth factor production as compared to the control (Figure 5C). Gene expression profiling of the M1 macrophages was carried out on cDNA analysis. Gene expression results showed similar trends to the ELISA assay. Hypermongone C downregulated the pro-inflammatory gene expression of IL-6 by 50% (Figure 5D). However, TNF-α gene expression remained the same as compared to the control (Figure 5E). Meanwhile, the gene expression of VEGF increased 2-fold compared to the control (Figure 5F). For each sample, we normalized the expression ratios to the reference primer glyceraldehyde 3-phosphate dehydrogenase GAPDH (housekeeper) gene, presenting the results as relative values. The promising upregulation of VEGF suggests hypermongone C’s ability to promote endothelial cell sprouting and tube formation in vivo. Similarly, the suppression of transcription factors TNF-α and IL-6 indicates that the compound can attenuate inflammation.

## 3. Discussion

This study reports for the first time that hypermongone C, a PPAP isolated from *H. scabrum*, accelerates wound healing and wound closure in vitro, modulates the immune response, and induces tube formation on matrigel. We demonstrate that hypermongone C accelerates fibroblast migration into the gap, which mimics wound closure in vivo, reduces pro-inflammatory markers (IL-6 and TNF-α), and promotes endothelial cell proliferation and tube formation on reduced growth factor matrix.

Many plant-derived metabolites can accelerate wound healing and skin regeneration [31,32]. Medicinal plants and natural compounds have been traditionally used in treating wounds, and many in vitro and in vivo studies have proven their anti-oxidant, anti-bacterial, anti-inflammatory, and wound healing effects [33,34,35]. Several *Hypericum* species have been used for traditional wound healing and are considered a rich source of biologically active polycyclic structures demonstrating wound healing activity as well as other biological properties. However, these compounds are not well studied. Suntal et al. demonstrated that the ethanolic extract of *Hypericum scabrum* did not show wound healing effects using in vivo excision and incision wound models as compared to *Hypericum perforatum* [36]. Our results are different in that we demonstrated the promising in vitro wound healing potential of hypermongone C, as a pure compound isolated from the hexane extract of *Hypericum scabrum,* and not an extract. Moreover, the extracting method and polarity of solvents used in isolation techniques have a strong effect on the biological activity of the extract and pure compounds. This will lead to a different biological function. The yield of hypermongone C isolated from Hexane extract was measured at 0.02%. Although the yield is not that high, the plant is readily available in abundance in various parts of the world. Also, given the high activity level of this compound as compared to other extracts in the same group, we only need low concentrations to be applied in wound healing applications. These together demonstrate the feasibility of use in future clinical applications

Wound healing is a multistep, overlapping process controlled by the cooperation of different cells in a timely manner, and any failure of these interactions will result in chronic wounds and delayed healing [37]. Common chronic wounds and non-healing ulcers, such as diabetic foot, pressure, and venous leg ulcers, remain in the inflammatory phase. IL-6 and TNF-α proinflammatory factors are amplified in chronic non-healing ulcers, postponing the healing process [38]. Of great interest are treatments that can regulate the macrophage inflammatory phase to stimulate the successful transition to the tissue formation and repair phases [39,40].

We evaluated the immunomodulatory effect of hypermongone C using a 24-h treatment of the compound at 1 µg/mL on M1 macrophages polarized by LPS/IFN-γ. LPS is a bacterial component that activates the signaling pathway of nuclear factor-kappa B (NF-kB) to trigger the secretion of pro-inflammatory markers [41]. Our results showed that hypermongone C suppresses the secretion of pro-inflammatory markers TNF-α and IL-6. This is consistent with the anti-inflammatory effect of hypermongone G, which features a similar structure to hypermongone C and demonstrates a significant inhibitory effect on nitric oxide production in LPS-induced RAW264.7 macrophages [30].

We determined the mechanism under which hypermongone C exerts its immune modulating effect through the analysis of the inflammatory marker secretions of M1 macrophages at the gene and protein level. We observed reduced expression of IL-6 at both protein and gene levels in hypermongone C-treated cells. Additionally, TNF-α expression was suppressed at the protein level compared to the untreated cells, while there was no change in TNF-α reduction at gene level. Gene expression is controlled and regulated at different stages, such as transcription and post translation [42]. Transcriptional cell activity does not always correlate to protein synthesis. Amsen et al. reported that the presence of RNA does not necessarily reflect protein levels. For example, some cytokines (IL-4 and IL-10) are regulated at the translational level, and others such as IL-1 and IL-18 at the post-translational stage [43]. These observations suggest the anti-inflammatory properties of hypermongone C. This is consistent with other studies that demonstrated compounds isolated from *Hypericum* genus possess potent anti-inflammatory effects [24,25,44].

The other essential factor associated with late healing and wound contraction is collagen formation, which is in control of dermal and epidermal cells. We performed an in vitro scratch assay on fibroblasts to investigate the proliferation and migration of these cells treated with hypermongone C under physiological conditions. We found that hypermongone C accelerates fibroblast cell migration and gap closure (almost two-folds faster than the control), which indicates the stimulatory effect of this compound. Traditionally, growth factors have been utilized to promote this stage of wound healing [3]. In light of these new exciting findings, we believe that hypermongone C can be used alone or in combination with growth factors to accelerate the process.

The proper proliferation and migration of fibroblasts will lead to the formation of granulation tissue, which requires vascularization to support cellular growth and tissue regeneration [45] Vascularization is amplified by inflammation and is suppressed at the late stage of healing when tissue is formed, and inflammation is reduced. As a result, there is a close relation between delayed healing and impaired vascularization [19,46]. The process of endothelial cell proliferation, migration, and angiogenesis is considered one of the most important steps in wound healing, which takes place from the beginning to the end of the healing process. Hypermongone C at the optimum concentration of 1 µg/mL induced endothelial cell proliferation and tube formation on a matrigel substrate. In contrast, we observed fewer tubes formed in the untreated cells, with many of them remaining individual, single cells. Interestingly, the enhanced expression of the VEGF gene involved in vascularization confirmed the angiogenic stimulatory potential of hypermongone C. VEGF upregulation at the early stage of healing can shift the process to the second phase more quickly. Also, VEGF is a vital component of vascularization in the process of healing, aiding in the transfer of nutrients and oxygen to the forming tissue [18].

The main inspiration behind this work was the extraordinary characteristic of natural compounds to target multiple molecular mechanisms in the immune system and regulatory pathways. A single compound with bioactive moieties that can simultaneously modulate multiple signaling transduction is rare to find. To this end, this paper serves as a pioneering attempt to identify a natural compound with the potential to modulate the interaction between various biological activators of wound regeneration. Further studies are warranted to validate the in vivo efficacy of this compound prior to clinical use.

## 4. Materials and Methods

### 4.1. Hypermongone C Source and Identification

Hypermongone C was isolated from the aerial part of *H. scabrum* using chromatographic techniques. The dried aerial parts of *H. scabrum* were extracted with hexane. The dried hexane extract (150 g) was subjected to silica gel column chromatography and was eluted with a gradient of non-polar to polar solvents. The column effluent was collected to afford 8 fractions. Fraction 2 (3 g) was further separated on a silica gel column, followed by preparative and semi-preparative RP-HPLC (Knauer, Berlin, Germany) (H_2_O/MeCN, 20:80) to yield hypermongone C. Isolation was performed using Knauer HPLC system and on a RP C18 (5 µm, 4.6 × 250 mm i.d) and Sunfire C18 (5 µm, 19 × 50 mm i.d). UV spectra was recorded from 210 to 400 nm. The yield of hypermongone C isolated from hexane extract was measured at 0.02%. The structure of the compound (Figure 1) was elucidated by using 1D and 2D-nuclear magnetic resonance (NMR) spectroscopy (Bruker, Billerica, MA, USA). ^1^H-NMR, H-H correlation spectroscopy (COSY), heteronuclear single quantum coherence (HSQC), and heteronuclear multiple bond correlation (HMBC) were performed for structural determination (Figure S1). NMR spectra were recorded in dimethyl sulfoxide (DMSO-*d*_6_) using a Bruker Avance III-HD 400 MHz. The molecular formula was determined by time of flight mass spectrometry (TOF-MS) (Figure S2). The TOF-MS spectrum of hypermongone C was recorded in methanol using the positive ion mode on an Orbitrap Velos Pro mass spectrometer (Thermo Fisher, Waltham, MA, USA).

### 4.2. Cell Culture and Reagents

Human umbilical vein endothelial cells (HUVECs), primary human dermal fibroblasts (HDFs), and related media for cell culture, including endothelial growth medium, were purchased from Lonza (Walkersville, MD, USA). HDFs were cultured in Dulbecco’s modified Eagle’s medium (DMEM/F12) (Corning, NY, USA) supplemented with 10% fetal bovine serum (FBS) (FB Essence, VWR, PA, USA) and 1% penicillin/streptomycin (HyClone, IL, USA) and incubated at 37 °C and 5% CO_2_ throughout the experiment. THP-1 human monocytic cells were obtained from American Type Culture Collection (ATCC) (Manassas, VA, USA). THP-1 cells were maintained in Roswell Park Memorial Institute (RPMI-1640) medium (Corning) supplemented with 10% fetal bovine essence (FBE; VWR, USA) and 0.05 mM 2-mercaptoethanol (Sigma-Aldrich, Milwaukee, WI, USA). Phosphate-buffered saline (PBS) and [3-(4,5-dimethylthiazol-2-yl)-5-(3-carboxymethoxyphenyl)-2-(4-sulfophenyl)-2*H*-tetrazolium (MTS) colorimetric assay were purchased from Promega (Madison, WI, USA), while growth factor reduced matrigel BD was from Corning. The cell staining solution was obtained from Cell Biolabs (San Diego, CA, USA) and Culture-Insert was purchased from IbiTreat (Martinsried, Germany). 12-myristate 13-acetate (PMA) and LPS from Escherichia Coli were purchased from Sigma. Interferon-gamma (IFN-γ) was purchased from PeproTech (Rocky Hill, NJ, USA).

### 4.3. Cytotoxicity Assay (MTS)

In order to determine cell viability, HUVECs and HDFs were cultured in their growth media to reach 80% confluency. Cells were seeded at a density of 5 × 10^3^ cell per 96 well plates in a total volume of 100 µL in each well. After seeding, the cells were incubated for 24 h at 37 °C and 5% CO_2_ to allow for cell attachment. Media supplemented with the different concentrations of hypermongone C (0.1, 1, and 10 µg/mL) was replaced. The compound was first dissolved at a concentration of 10 mg/mL in DMSO and subsequently diluted in culture media. Following 24 h incubation, media containing 20% MTS solution was replaced with growth media and incubated for 2 h. The absorbance of each well at 490 nm was measured using a Spectramax 190 spectrometer (Sunnyvale, CA, USA).

### 4.4. LIVE/DEAD^®^ Assay

The LIVE/DEAD^®^ viability/cytotoxicity kit (Thermo Fisher, Waltham, MA, USA) was used to visually analyze the viability of the HUVECs and HDFs following 8 and 24-h exposure to different concentrations of hypermongone C. This method discriminates live from dead cells by simultaneously staining with green–fluorescent calcein-AM to indicate intracellular esterase activity and red-fluorescent ethidium homodimer-1 to indicate loss of plasma membrane integrity. Cells with a density of 5 × 10^3^ were seeded in a 96 well plates. Cells with 80% confluency were supplemented with different concentrations of hypermongone C (0.1, 1, and 10 µg/mL) in growth media. Following appropriate incubation, cells were washed with PBS, then treated with LIVE/DEAD^®^ solution for 15 min. After washing in PBS, cells were imaged using a fluorescence Nikon Eclipse Ti-E inverted microscope (Melville, NY, USA).

### 4.5. In Vitro Migration Assay (Wound Healing Assay)

The cell migration of HDFs was assessed using a Culture-insert, consisting of two wells that were separated by a wall. A total of 70 µL of cell suspension comprised of 35 × 10^3^ cells was cultured in each well. Following 24-h attachment and full confluency, the culture inserts were removed to form a cell-free gap. Cells were washed with PBS to remove cell debris and then supplemented with different concentrations of hypermongone in growth media and incubated at 37 °C and 5% CO_2_ for 22 h. Images were taken at different time intervals using a phase contrast Nikon Eclipse Ti-E inverted microscope. Quantification of the percentage wound healing was calculated by measuring the gap distance using the following formula:

Wound closure % = (W0−Wn)/W0×100%

in which *Wn* is the width of the gap after different time intervals and *W*0 is the initial width right after forming a scratch. Cells at the zero and 22 h time points were stained using cell stain solution. The media of the cells was removed and 400 µL of cell stain solution was added to each well. Cells were incubated for 15 min at room temperature. Images were taken at different time intervals using a phase contrast inverted microscope (Invitrogen EVOS FL Auto Cell Imaging, Waltham, MA USA).

### 4.6. Capillary Tube Formation

Growth factor reduced BD matrigel was kept in a −20 °C freezer and thawed on ice overnight in a 4 °C refrigerator. To form a gel, 50 µL of the thawed matrigel was added to each well of a pre-chilled 96-well plate and then incubated for 30 min at 37 °C. 100 µL of a HUVEC cell (cell passages: 2–6) suspension in endothelial growth media (20,000 cells/well), including different concentrations of hypermongone C (0.1 and 1 µg/mL), was added to the gel and incubated at 37 °C for 8 h [47]. The number of tubes was examined using a phase contrast inverted microscope (Invitrogen EVOS FL Auto Cell Imaging) and compared to the condition with no compound (negative control).

### 4.7. Macrophage Polarization

THP-1 cells with a density of 1 × 10^6^ cells/well with a total volume of 2 mL of culture media were differentiated into M0 macrophages by culturing the cells with 100 ng/mL of PMA for 24 h. After differentiation, the cells were washed three times with serum-free media RPMI-1640 (Gibco) to remove non-differentiated cells. To polarize M0 macrophages to M1 macrophages, the cells were exposed to culture media supplemented with 100 ng/mL of LPS and 20 ng/mL IFN-γ for 24 h.

### 4.8. Enzyme-Linked Immunosorbent Assay (ELISA)

M0 macrophages were polarized to M1 macrophages in media containing hypermongone C and the polarizing agents including IFN-γ and LPS. Following 24 h incubation, the M1 media was collected, centrifuged, and stored at −20 °C for further experiments. The culture media from the M1 macrophages was analyzed to determine the cytokine concentrations of TNF-α, IL-6, and VEGF using an ELISA assay according to the manufacturer’s protocols (PeproTech). Colorimetric changes were measured using a SpectraMax 190 microplate spectrophotometer at 450 nm with the wavelength correction set at 620 nm. Standard curves for each cytokine were run in parallel to convert the absorbance to concentration in each group.

### 4.9. RNA Extraction and Quantitative Real-Time Polymerase Chain Reaction (RT-PCR)

Gene Jet RNA Purification kit (Thermo Scientific) was used to isolate total ribonucleic acid (RNA) according to the manufacturer’s instructions. RNA was quantified on a Thermo Scientific Nanodrop 2000c Spectrometer and considered pure if the ratio of the absorbance at 260 nm/280 nm was ≥ 2. Then, samples were stored at −20 °C until they were analyzed for RT-PCR. The RNA was prepared as a template for complementary deoxyribonucleic acid (cDNA) synthesis using the iScript cDNA Synthesis kit (Bio-Rad). Quantitative RT-PCR analysis was performed using 10.4 ng of cDNA per reaction and SYBER^®^ Green PCR Supermix (Bio-Rad). Gene expression was normalized to the housekeeping gene GAPDH and the control group (2^−ΔΔC^). Gene expression values were calculated using the mean cycle threshold (CT) values of the samples. All primers (Table S2) were synthesized by Integrated DNA Technologies (Coralville, IA, USA).

### 4.10. Statistical Analysis

Three samples (*n* = 3) were analyzed per condition unless otherwise stated. All data were statistically presented as the mean ± standard error. Multiple *t*-tests were performed using Graph-Pad Prism 7.03 (La Jolla, CA, USA) to determine the significance between each experimental group. P values of less than 0.05 were significant.

## 5. Conclusions

In this study, we set out to simultaneously stimulate cell proliferation and migration as well as affecting macrophage function using a potent naturally derived compound. Results of this study showed that hypermongone C accelerates different phases of healing, including the proliferation and migration of fibroblasts, induction of endothelial cell tube formation and VEGF secretion, and the regulation of the immune markers IL-6 and TNF-α. Therefore, the wound-healing potential of hypermongone C, which we have tested here for the first time, offers a novel multi-targeting therapeutic that can be used alone or in combination with other therapies. We suggest that hypermongone C be used as a topical formulation or impregnated in wound dressings and applied on animal wound models to further investigate wound closure in vivo.

## Supplementary Materials

The following are available online, Figure S1: The (**A**) 1H-NMR, (**B**) H-H COSY, (**C**) HSQC, and (**D**) HMBC spectra of hypermongone C in DMSO-*d*_6_, Figure S2: High resolution mass spectrometry of hypermongone C in positive mode, Table S1: 1H and 13C-NMR assignment, Table S2: Primers used for quantitative real-time polymerase chain reaction.
