# Supplementary material for: Hypermongone C Accelerates Wound Healing through the Modulation of Inflammatory Factors and Promotion of Fibroblast Migration
Source: Molecules. 2019 May 27;24(10):2022. doi: 10.3390/molecules24102022 (PMC6572485; doi:10.3390/molecules24102022)
Supplement: Supplementary file 1 [file molecules-24-02022-s001.pdf]

Hypermongone C accelerates wound healing through the modulation of inflammatory factors and promotion of fibroblast migration

Sara E. Moghadam [1], Mahdi Moridi Farimani [2], Sara Soroury[3], Samad N. Ebrahimi[2], and Ehsan Jabbarzadeh [1,4,*]

[1] Department of Chemical Engineering, University of South Carolina, Columbia, SC, 29208, USA; eslambol@mailbox.sc.edu
[2] Department of Phytochemistry, Medicinal Plants and Drug Research Institute, Shahid Beheshti University, GC, Evin, Tehran, 1983969411, Iran; m_moridi@sbu.ac.ir; s_ebrahimi@sbu.ac.ir
[3] Department of Phytochemistry, Faculty of Science, Golestan University, Gorgan, 49138157559, Iran; sarasoroury@gmail.com
[4] Biomedical Engineering Program, University of South Carolina, Columbia, SC, 29208, USA; ehsan@sc.edu
* Correspondence: ehsan@sc.edu, Tel.: (803) 777-3297

Supplementry Data

(A)

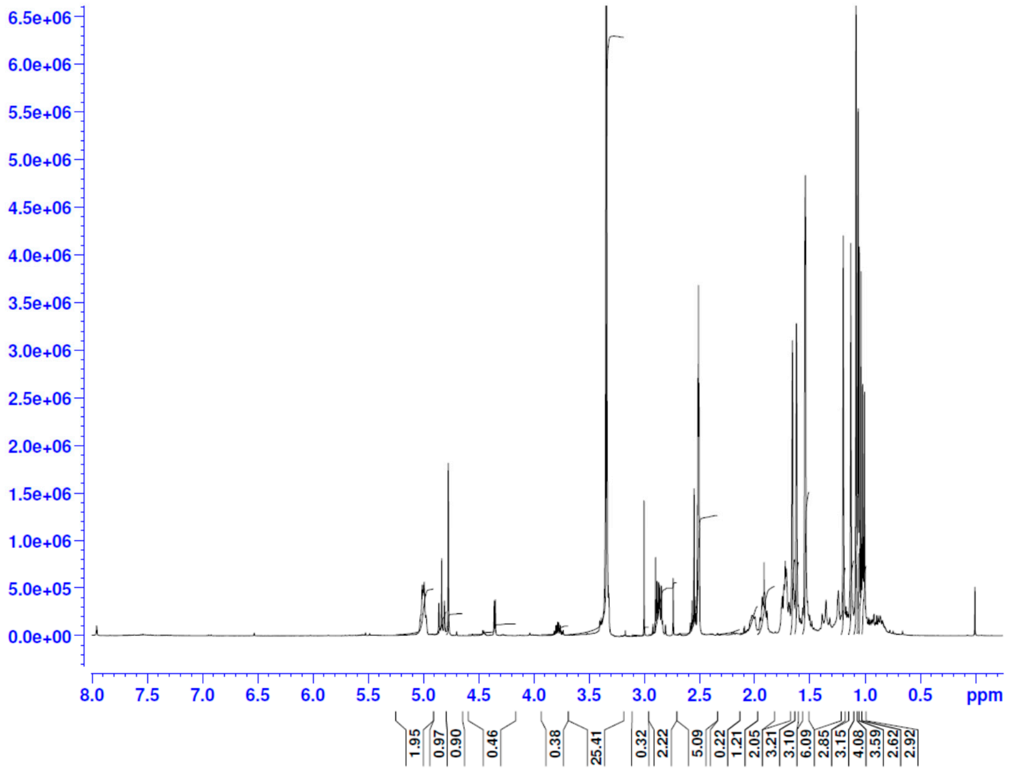

(B)

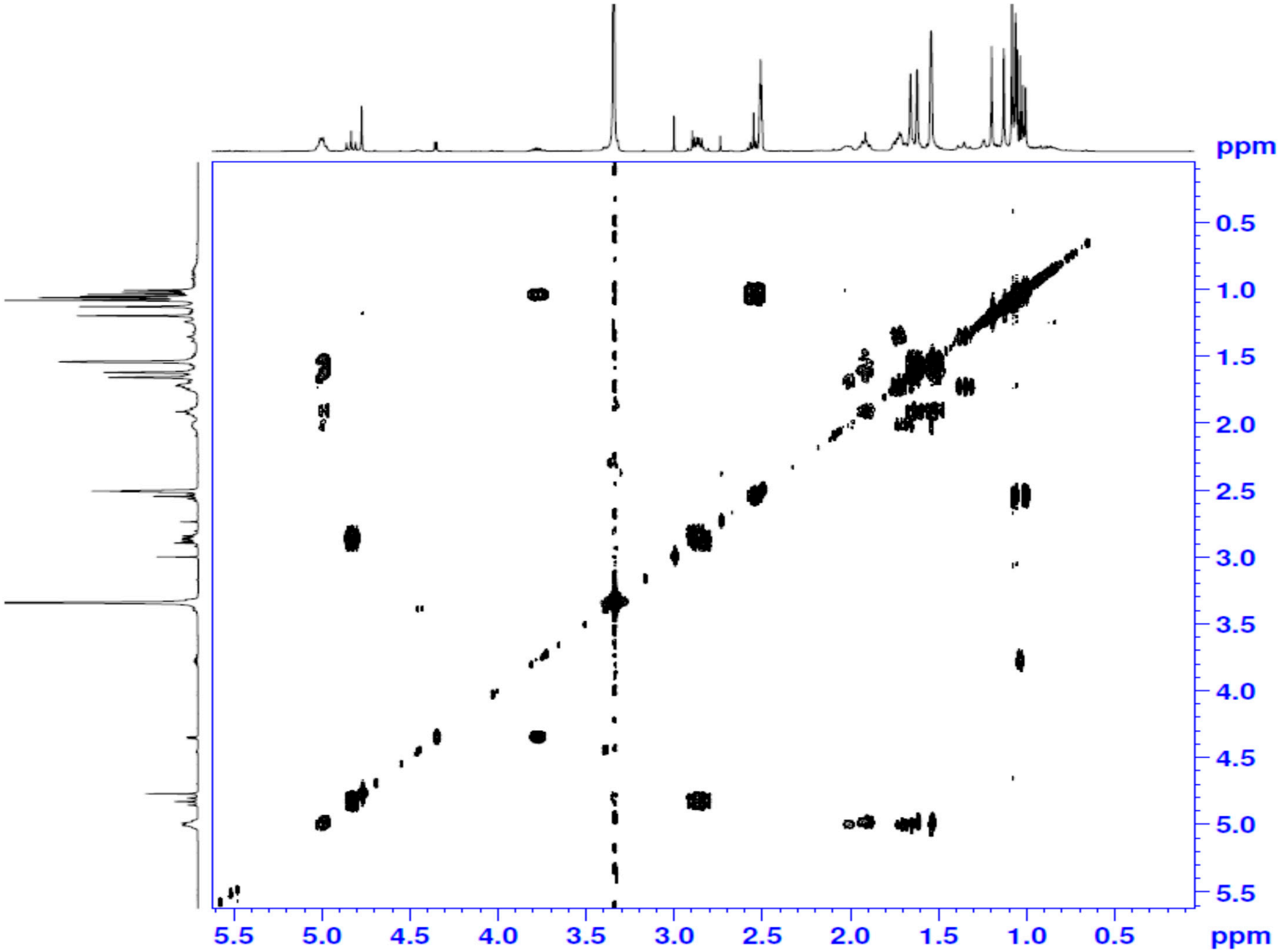
ppm
0.5
1.0
1.5
2.0
2.5
3.0
3.5
4.0
4.5
5.0
5.5
5.5
5.0
4.5
4.0
3.5
3.0
2.5
2.0
1.5
1.0
0.5
ppm

(C)

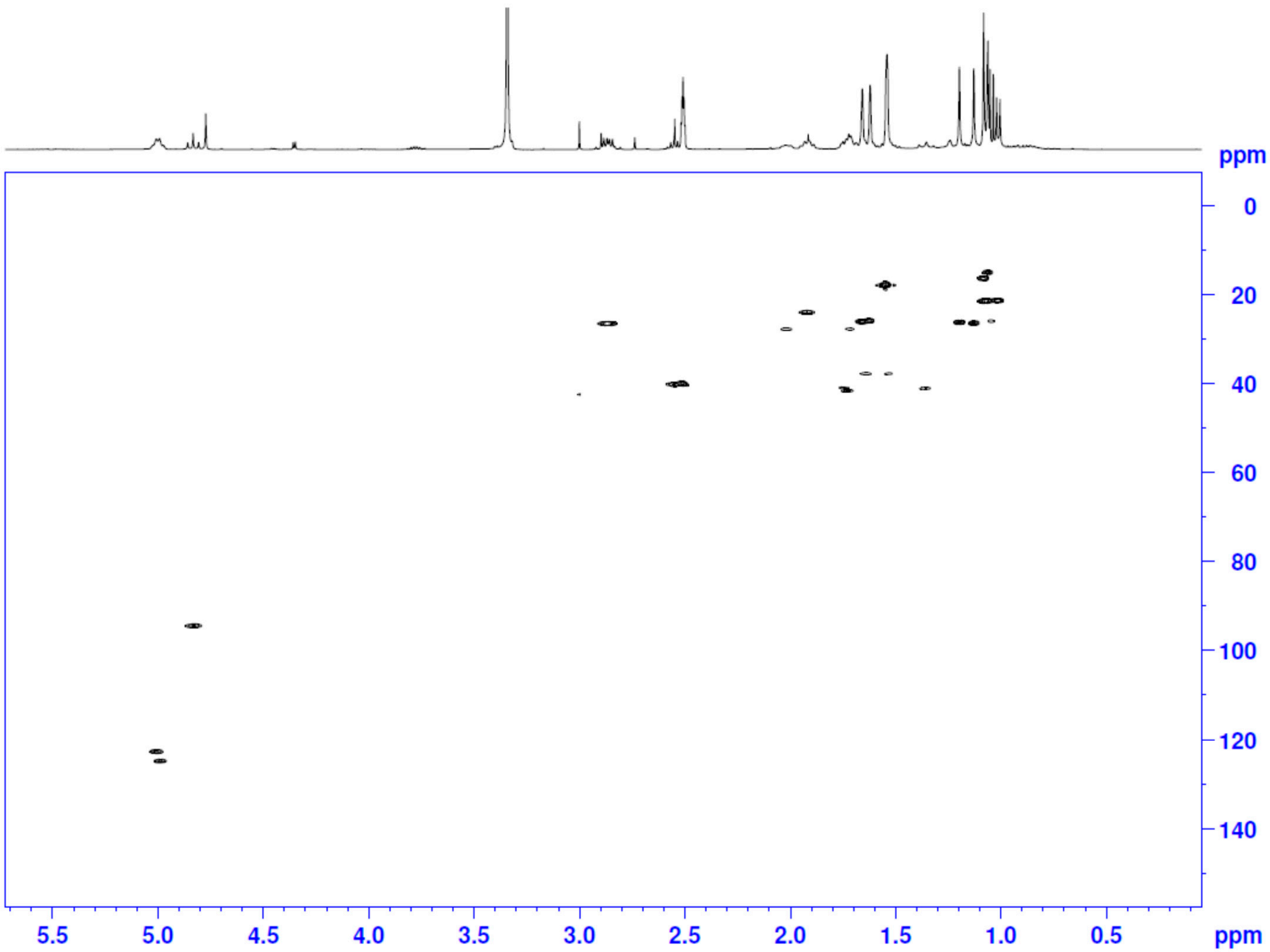

(D)

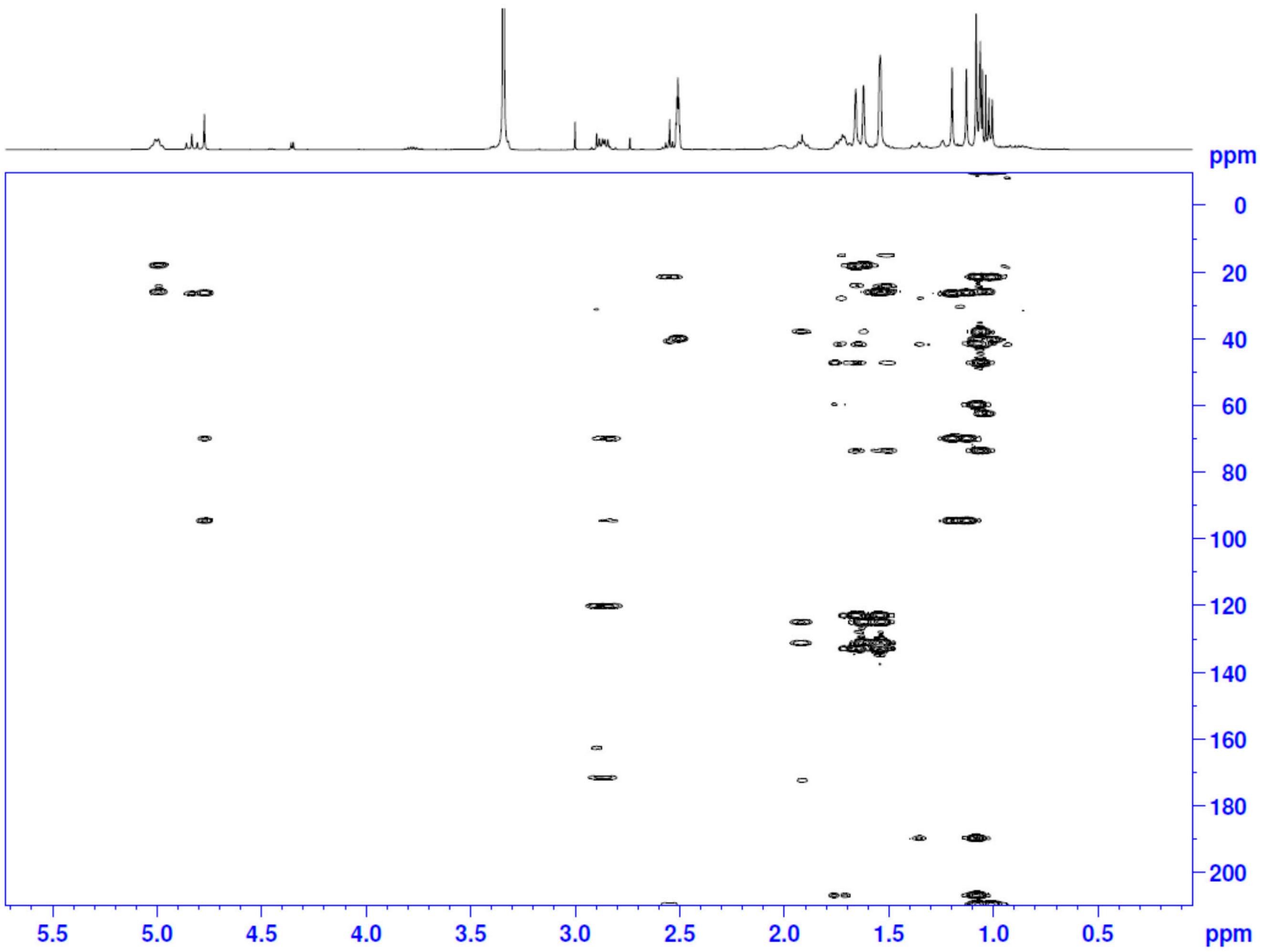

Figure S1. The (A) ^{1}H-NMR, (B) H-H COSY, (C) HSQC, and (D) HMBC spectra of Hypermongone C in DMSO-d_6.

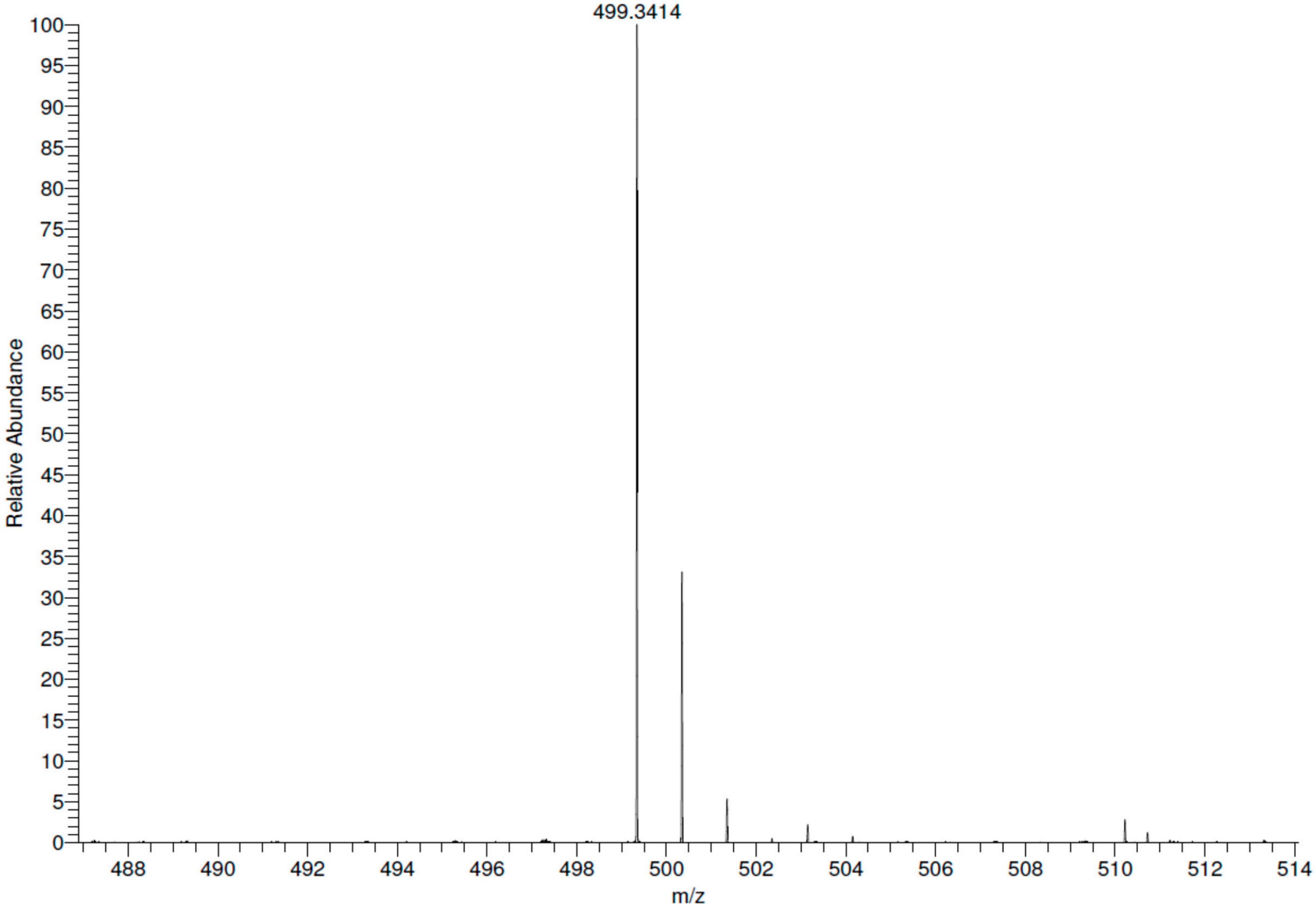

Figure S2. High resolution mass spectrometry of Hypermongone C in positive mode.

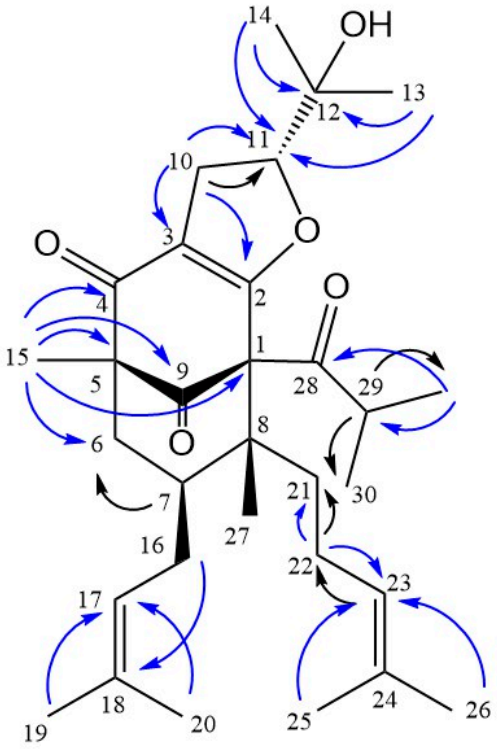

Figure S3. H-H COSY (⟶) and HMBC (⟶) correlations

Table S1. 1H and 13C NMR data (400 and 100 MHz, in DMSO-d6) of compound hypermongone C

position	δ_H	δ_C	position	δ_H	δ_C
1	-	73.6	31	1.01 (d)	20.6
2	-	171.7			
3		120.2			
4		189.8			
5		59.7			
6a	1.74 (dd)	41.1			
6b	1.36 (t)	41.1			
7	1.73 (m)	41.8			
8		47.3			
9		207.0			
10a	2.89 (dd)	26.6			
10b	2.84 (dd)	26.6			
11	4.80 (t)	93.5			
12		70.1			
13	1.12 (s)	25.9			
14	1.19 (s)	26.1			
15	1.08 (s)	16.4			
16a	2.02 (m)	27.8			
16b	1.72 (m)	27.8			
17	4.9 (m)	124.9			
18		133.0			
19	1.54 (bs)	18.0			
20	1.63 (s)	25.9			
21a	1.64 (m)	37.9			
21b	1.53 (bs)	37.9			
22a	1.93 (m)	24.0			
22b	1.90 (m)	24.0			
23	5.00 (m)	122.0			
24		131.3			
25	1.54 (bs)	18.0			
26	1.66 (s)	25.9			
27	1.06 (s)	15.0			
28		209.0			
29	2.55 (m)	40.3			
30	1.08 (d)	20.6			

Table S2. Primers used for quantitative real-time polymerase chain reaction.

Gene	5'-3' primer sequences: (F: forward R: reverse)
TNF-α	F: CTG CTG CAC TTT GGA GTG AT
	R: AGA TGA TCT GAC TGC CTG GG
IL-6	F: AGC CAC TCA CCT CTT CAG AAC
	R: GCC TCT TTG CTG CTT TCA CAC
VEGF-A	F: AGC CTT GCC TTG CTG CTC TA
	R: GTG CTG GCC TTG GTG AGG
GAPDH	F: GTG GAC CTG ACC TGC CGT CT
	F: GGA GGA GTG GGT GTC GCT GT
